# Neuroprotection and immunomodulation by xenografted human mesenchymal stem cells following spinal cord ventral root avulsion

**DOI:** 10.1038/srep16167

**Published:** 2015-11-09

**Authors:** Thiago B. Ribeiro, Adriana S. S. Duarte, Ana Leda F. Longhini, Fernando Pradella, Alessandro S. Farias, Angela C. M. Luzo, Alexandre L. R. Oliveira, Sara Teresinha Olalla Saad

**Affiliations:** 1 Hematology and Hemotherapy Center-University of Campinas/Hemocentro-Unicamp, Instituto Nacional de Ciência e Tecnologia do Sangue, Campinas, São Paulo, Brazil; 2 Neuroimmunomodulation Group, Dept. Genetics, Evolution and Bioagents, University of Campinas, Campinas, Brazil; 3 Dept. of Structural and Functional Biology, Institute of Biology, University of Campinas, Campinas, Brazil

## Abstract

The present study investigates the effects of xenotransplantation of Adipose Tissue Mesenchymal Stem Cells (AT-MSCs) in animals after ventral root avulsion. AT-MSC has similar characteristics to bone marrow mesenchymal stem cells (BM-MSCs), such as immunomodulatory properties and expression of neurotrophic factors. In this study, Lewis rats were submitted to surgery for unilateral avulsion of the lumbar ventral roots and received 5 × 10^5^ AT-MSCs via the lateral funiculus. Two weeks after cell administration, the animals were sacrificed and the moto neurons, T lymphocytes and cell defense nervous system were analyzed. An increased neuronal survival and partial preservation of synaptophysin-positive nerve terminals, related to GDNF and BDNF expression of AT-MSCs, and reduction of pro-inflammatory reaction were observed. In conclusion, AT-MSCs prevent second phase neuronal injury, since they suppressed lymphocyte, astroglia and microglia effects, which finally contributed to rat motor-neuron survival and synaptic stability of the lesioned motor-neuron. Moreover, the survival of the injected AT- MSCs lasted for at least 14 days. These results indicate that neuronal survival after lesion, followed by mesenchymal stem cell (MSC) administration, might occur through cytokine release and immunomodulation, thus suggesting that AT-MSCs are promising cells for the therapy of neuronal lesions.

Research with embryonic and adult stem cells has been undertaken these past recent years and has been rendering interesting results in the field of regenerative medicine^1^. Mesenchymal stem cells (MSCs) may have a promising use in cellular therapies for various diseases: graft-versus-host disease (GVHD)^2^,^3^, autoimmune diseases^4^,^5^ and neurological diseases^6^,^7^. Many studies have demonstrated the positive effect of MSCs in several animal models: autoimmune encephalomyelitis (EAE)^8^,^9^, diabetes^10^, cerebral ischemia^11^ and spinal cord injuries^12^,^13^,^14^.

For clinical purposes, protocols have been designed for efficiently obtaining and using AT-MSCs^15^,^16^,^17^, however, there is a fundamental concern regarding the safety of the therapeutic use of MSCs for allogeneic transplantation. Studies using human MSCs in xenotransplant of several animal models may help clarify the functions and effects of these cells on a non-self-environment. Xenotransplantation is defined by intraspecies and interspecies transplantation in immunocompetent animals with no immunosuppressive drugs^18^. As this type of study raises many issues regarding functionality, durability and effects of MSCs in the animal’s body, the setting of safety standards is essential.

In the herein work, ventral root avulsion (VRA) at the spinal cord surface induced in Lewis rats, which mimics the brachial plexus injury in humans, was used to investigate the effects of human AT-MSCs xenotransplantation. In this model, injuries to spinal motor neurons in the interface between the central and peripheral nervous system result in a neuronal loss of up to 80% during the first two weeks^19^. The lesioned site becomes an inflammatory microenvironment favoring the sudden activation of resident glial cells, contributing to acute homeostasis changes and loss of synaptic inputs^20^. The lesion mimics the brachial plexus injury in humans, the most common avulsion injury resulting from motorcycle accidents, leading to motor, sensory or/and autonomic loss of the affected extremity^21^.

Despite MSCs differentiation capacity being well described *in vitro*, the paracrine action of AT-MSCs has been demonstrated in many intraspecies-transplantation models. Animal AT-MSC transplanted in cardiac repair models exhibited angiogenesis and reduction in cardiomyocyte apoptosis at the infarct border zone^22^. Lopatina *et al.* showed that transplanted cells induced nerve repair and growth via BDNF production following cell xenotransplantation in mice limb re-innervation-models^23^. Wei *et al.* further confirmed that medium secreted by AT-MSC avoided neuronal apoptosis, supporting the hypothesis that AT-MSC direct delivery could have therapeutic use in neurodegenerative disorders^24^,^25^. Treatment of experimental autoimmune diabetes suggests that AT-MSC transplantation could improve autoimmune diabetes pathogen by attenuating Th1 immune response simultaneous to Tregs expansion/proliferation^26^. Other authors have also shown the effects of AT-MSC xenotransplantation in several animal models: as promotion of angiogenesis and cell survival, immunosuppression effects and others^14^,^27^,^28^. Mesenchymal stem cells derived from adipose tissue (AT-MSCs) do not express major histocompatibility complex antigen II (MHC II)^29^ and might exert immunomodulatory action throughout cytokine release (TGF-β, HGF, prostaglandin E2 and other soluble factors)^9^,^30^,^31^,^32^ and indoleamine 2,3-dioxygenase (IDO)^33^. Mesenchymal stem cells(MSCs) have been used in clinical trial phase 1, establishing the safety of clinical application of MSCs, however further modifications to improve MSCs efficacy are required^18^,^34^,^35^,^36^,^37^.

In this study, we aimed to verify whether human AT-MSCs exert neuroprotective and immunomodulatory effects upon a xenograft model of ventral root avulsion (VRA). Our results demonstrated that human AT-MSCs locally suppress the rat immune system by reducing T cells and resident glia reactivity in the affected area and increase motor neuron survival through neuroprotective mechanisms.

## Material and Methods

The methods described herein were carried out in accordance with the approved guidelines. The Ethics Committee of the Faculty of Medical Sciences and Institute of Biology of the University of Campinas approved all experimental protocols.

### Isolation and culture of mesenchymal stem cells from adipose tissue (AT-MSCs)

AT-MSCs cultures were established from AT obtained from seven healthy patients submitted to lipoaspiration procedures. All donors signed a free informed consent and all procedures were approved by the Ethics Committee of the Faculty of Medical Sciences, University of Campinas. AT-MSCs isolation was performed as previously described^38^ and incubated in DMEM/ 10% FBS at 37 °C in a humidified atmosphere with 5% CO2. At 80% confluence, AT-MSCs were detached with 0.25% trypsin −0.02% EDTA and replated at a lower density. All tissue culture reagents were purchased from InvitrogenTM Life Technologies – New York, NY.

### AT-MSCs characterization and Qtracker labeling

At the third passage, immunophenotypical analysis of AT-MSCs-was performed using FITC-, PE-, or PECy5 – conjugated monoclonal antibodies (mAbs) against CD90, CD105, CD73, CD29, CD45, CD34, HLA-DR and HLA-ABC and their respective isotype control mAbs (BD Biosciences, Mountain View, CA, USA). Briefly, AT-MSCs were resuspended at a concentration of 10^6^ cells/ml, incubated ABs at 4 °C for 30 min. AT-MSCs were then washed and analyzed by flow cytometry using FACS Calibur and CellQuest software (10.000 events/sample - BD Biosciences, San Jose, CA, USA). AT-MSCs were submitted to differentiation protocols for multi-potential differentiation capacity verification. AT-MSCs were cultured under specific differentiation medium for 21 days and confirmed by specific cell staining for each differentiation (Table 1).

Prior to *in vivo* injection, AT-MSCs within 3–5 passages were marked with QTracker 655 (Invitrogen) according to the manufacturer’s protocol for cell identification and location after grafting.

### RT-PCR

Gene expressions were detected by reverse transcriptase-polymerase chain reaction (RT-PCR). Total RNA was extracted from AT-MSCs with illustra RNAspin Mini isolation RNA Kit according to manufacturer’s protocol (GE Healthcare Life Siences - USA). Using total RNA as template, reverse transcription reactions were performed with RevertAid First Strand cDNA Synthesis Kit (Thermo Scientific) using oligo dT-adaptador primer according to the manufacturer’s protocol. PCR amplification was then performed with 200 nM of primers for human TGF-β1, HGF, IDO, GDNF, BDNF and β2-microglobulin (Table 2). PCR cycles were: 94 °C for 5 minutes, (94 °C for 30 seconds, 55 °C for 30 seconds, 72 °C for 45 seconds) × 35 cycles, 72 °C for 5 minutes. PCR products were analyzed by electrophoresis on 1.5% agarose gel and image acquisition and data analysis were accomplished with Transluminator L-Pix (Loccus Biotecnologia).

### Proliferative response of MBP-specific T lymphocytes

MBP-specific T lymphocytes (1 × 10^6^, initial input) plus APCs from thymus were cultured in the presence of their specific antigen (10 μg/ml) with or without AT-MSCs. After 3 days of culture, MBP-specific T lymphocytes were stained with trypan blue and counted in a TC10 automated cell counter (BioRad, USA).

### Surgery, transplantation and cell tracing

Rats (n = 5) were subjected to unilateral avulsion of the lumbar ventral roots as previously described^39^. Immediately after injury (up to 5 minutes), QTracker655 labeled AT-MSCs were placed directly into the lateral lesioned side funiculus (ipsilateral) in segments L4–L6 of the spinal cord at three equidistant points along the lesioned segment using a thick capillary Pasteur pipette coupled to a Hamilton syringe (Supplement Fig. 1A). The spinal cord was then replaced in its original position and musculature, fascia and skin sutured in layers.

Animals were sacrificed 14 days after surgery and fixed by vascular perfusion with 4% formaldehyde in phosphate buffer (pH 7.4). Lumbar enlargement was then dissected, post-fixed overnight in the same fixative and frozen. Cryostat transverse sections (12 μm) of spinal cords were obtained and transferred to silane-coated slides.

### Neuronal survival

Images for counting neurons were acquired by Nikon Eclipse E600 microscope equipped with light, epifluorescence illumination and high-resolution CCD camera (RT Slider, Diagnostic Instruments, Inc., Sterling Heights, MI, USA) and PC running Image-Pro software (Media Cybernetics, Inc., Silver Spring, MD, USA). Silane-coated slides were stained for 5 to 10 min in an aqueous 1% cresyl fast violet solution at 45 °C. Sections were then washed in distilled water and mounted in glycerol/PBS mixture (3:1).

Motor neurons were identified based on their morphology and location in the ventral horn (dorsolateral lamina IX). Only cells with a visible nucleus and nucleolus were counted. Counts were carried out on 20 sections (every fourth section) along the lumbar enlargement both on the ipsilateral and contralateral sides of each rat (n = 5). The absolute numbers of motor neurons on the lesioned and non-lesioned sides per section, respectively, were used to calculate the surviving cell percentage in each specimen. To correct for double counting of neurons, Abercrombie’s formula^40^ was used; **N = nt/(t + d)**, where *N* is the corrected number of counted neurons, *n* is the counted cell number, *t* is the section thickness (12 μm) and *d* is the average cell diameter. As size difference significantly affects cell counts, *d* was calculated specifically for each experimental group (ipsilateral and contralateral). In this regard, the diameter of the 15 randomly picked neurons from each group was measured (ImageTool software, version 3.00, The University of Texas Health Science Center Santo Antonio, USA) and the mean value calculated, as shown in Table 3.

### Immunohistochemistry

Primary anti-synaptophysin (1:200, Dako, Glostrup, Denmark), primary Anti-IBA1 (1:600, Wako, Osaka, Japan), primary anti-GFAP (1:100, Santa Cruz, Santa Cruz, CA, USA) and CD3-PE (1:200, Santa Cruz, Santa Cruz, CA, USA) were used to analyze synaptic covering, microglia activation, astroglial reaction and lymphocytes within the motor nucleus.

Primary and conjugated antibodies were diluted in a BSA and Triton x-100 in 0.01 M PBS solution. Sections were incubated overnight at 4 °C in a moist chamber. For primary AB, after rinsing in 0.01 M PBS, the sections were incubated with a Cy2-conjugated secondary antiserum (1:250, Jackson Immunoresearch, West Grove, PA, USA) for 45 min in a moist chamber at room temperature. Sections were then rinsed in PBS, mounted in glycerol/PBS mixture (3:1), and images were generated using a confocal laser-scanning microscope (LSM 510 Carl Zeiss). For quantitative measurements, three representative images of the ipsi and contralateral ventral horn were captured from each animal for all experimental groups, totalizing 15 sampled images from each side per group. Quantification was performed with the enhanced contrast and density-slicing feature of IMAGEJ software (version 1.33 u, National Institutes of Health, USA). Integrated density of pixels was systematically measured in six representative areas of the lateral motor nucleus from each side (lesioned and non-lesioned sides), according to Oliveira *et al.*^41^. The lesioned/non-lesioned ratio of the integrated density of pixels was calculated for each section and then as the mean value for each spinal cord. CD3 (Lymphocyte marker) detection was performed in sections of rats (n = 3) and in regions of the spinal cord laminae in which AT-MSCs were not present, according to absence of qdot 655 fluorescence.

### Statistical analysis

Data were analyzed using a two-tailed Student t test for parametric data or a two- tailed Mann–Whitney U test for non-parametric data at p < 0.05 (*), p < 0.01(**) and p < 0.001 (***). Data were presented as mean ± standard deviation (SD).

## Results

### Mesenchymal Stem Cell Characterization

AT-MSCs antigen surface characterization was performed by flow cytometry (FACS) using a panel of antibodies. At the fourth passage, cells were plastic adherent presenting fibroblastic morphology and positive for surface markers as CD90, CD105, CD73, CD29 and HLA-ABC, and negative for CD34, CD45 and HLA-DR (Supplement Fig. 2A).

To describe the plasticity attribute of AT-MSCs population (Supplement Fig. 2B), three differentiation protocols were performed. For osteogenic differentiation, cells were stained by Alizarin Red S, which shows calcium deposits (Supplement Fig. 2C). Cells submitted to adipogenic differentiation showed small red cytoplasmic vesicles, stained by Oil Red O, reflecting the accumulation of neutral lipids and triglycerides in cytoplasmic vesicles of differentiated cells (Supplement Fig. 2D). AT-MSCs were induced into chondrogenic differentiation and differentiated cells produced glycosaminoglycan matrix stained with Alcian blue (Supplement Fig. 2E).

### Cytokine expression and immunomodulatory action of AT-MSCs: *in vitro* proliferation assay of T Lymphocytes

**Figure 1:**
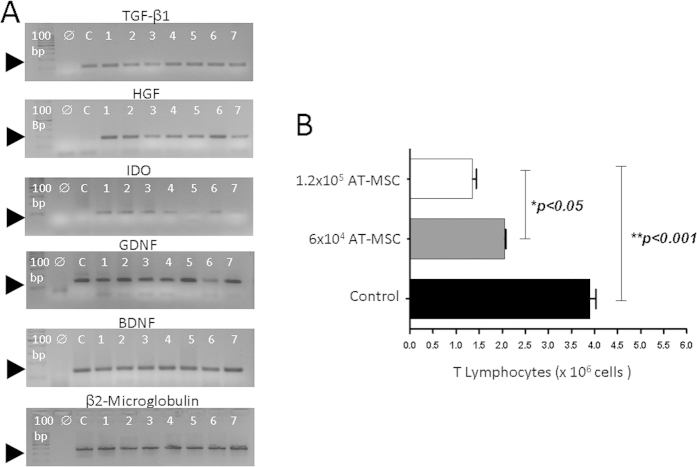
Immunosuppressive and neurotrophic major factors of AT-MSCs, during the 4th passage, and their *in vitro* effect on T cell proliferation. (**A**) Semi quantitative RT-PCR shows that AT-MSCs, from 7 donors, express mRNA immunosuppressive and neurotrophic factors as TGF-β1, HGF, IDO, GDNF and BDNF. A 100 bp ladder is shown in the first lane, the reaction using water instead of DNA is in the second lane. cDNA of Daudi cells was used as control (**C**). (**B**) MBP-specific T lymphocytes (initial input = 1 × 10^6^ T cells) plus Antigen Presenting Cells (APCs) from thymus were cultured in the presence of the myelin basic protein (MBP, black bar). Two different concentrations of AT-MSCs were added to the culture. Concentrations, 6 × 10^5^ (grey bar) and 1.2 × 10^6^ (white bar), induced a significant reduction in the proliferative response of MBP-specific T lymphocytes (n = 3).

Gene expression for neurotrophic factors (GDNF and BDNF) and immunosuppressive factors (TGF-β1, HGF and IDO) were analyzed for each cell sample (AT-MSCs) obtained from seven donors by semi-quantitative RT-PCR (Reverse transcription-polymerase chain reaction) (Fig. 1A). The endogenous control was used in the reaction: β2-microglobulin.Beta2-microglobulin gene transcripts demonstrated that samples had a similar expression pattern. BDNF and GDNF transcripts were also observed to be strongly expressed as TGFβ_1_ and HGF demonstrated that the expression of these genes maintained a similar level in all cell samples from different lipoaspirate tissue donors. IDO gene however, showed a variable expression level, suggesting that cell samples expressed the gene on a different level.

TGFβ1 and IDO expression by AT-MSCs suggested that these cells might present a tolerogenic function^42^. Therefore, we performed a T cell-specific proliferative assay to verify the influence of AT-MSCs over T cell activation and proliferation. Figure 1B shows that AT-MSCs reduced T cell specific proliferative response in a concentration dependent manner.

### AT-MSCs Grafting in the VRA model and AT-MSCs immunomodulatory effect in rat spinal cord

AT-MSCs were labeled with qdot655 (Invitrogen) and injected into the lateral funiculus of the spinal cord after lesion (Supplement Fig. 1A). After two weeks, animals were sacrificed and serial histological sections along the lesion in the spinal cord were performed. Labeled qdot655 cells were detected at the site of injection (Supplement Fig. 1B–D) confirming that human AT-MSCs survived for at least two weeks *in vivo*. Nonetheless, AT-MSCs were not identified in the gray matter region nor had they migrated to surrounding spinal cord areas. AT-MSCs apparently remained near the injection site.

**Figure 2:**
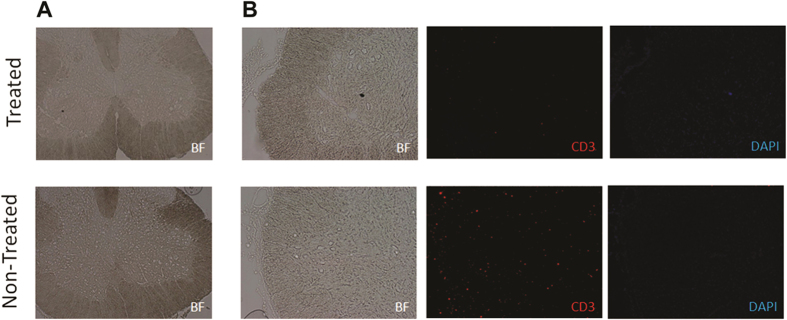
Paracrine effects of AT-MSCs *in vivo*. (The chosen section was AT-MSCs-free, according to qdot labeling analysis). Treated group showed lower presence of T lymphocytes compared with non-treated animals in lesioned area. (**A**) Spinal cord of treated and non-treated group (5×) (**B**) The section in both groups was marked with CD3 for Lymphocyte T detection and DAPI for nucleus detection (10×).

To investigate whether AT-MSCs had an *in vivo* paracrine immunomodulatory effect in the inflamed region, immunofluorescence with anti-CD3 was performed in regions of the spinal cord laminae in which AT-MSCs were not present, according to qdot 655 fluorescence absences. Excitingly, the CD3 marker in the rat spinal region was weakly positive in animals treated with AT-MSCs (Fig. 2A,B). In untreated animals however, the CD3 marker was present throughout the ventral spinal cord, demonstrating a strong T lymphocyte presence all along the affected area (Fig. 2B) and corroborating the T cell proliferation observed in the *in vitro* studies. Thus, the result suggests that AT-MSCs immunomodulatory profile acts upon the animal immune system decreasing inflammatory response.

### Neuroprotective effect of AT-MSCs on motor neurons and synaptic activity

**Figure 3:**
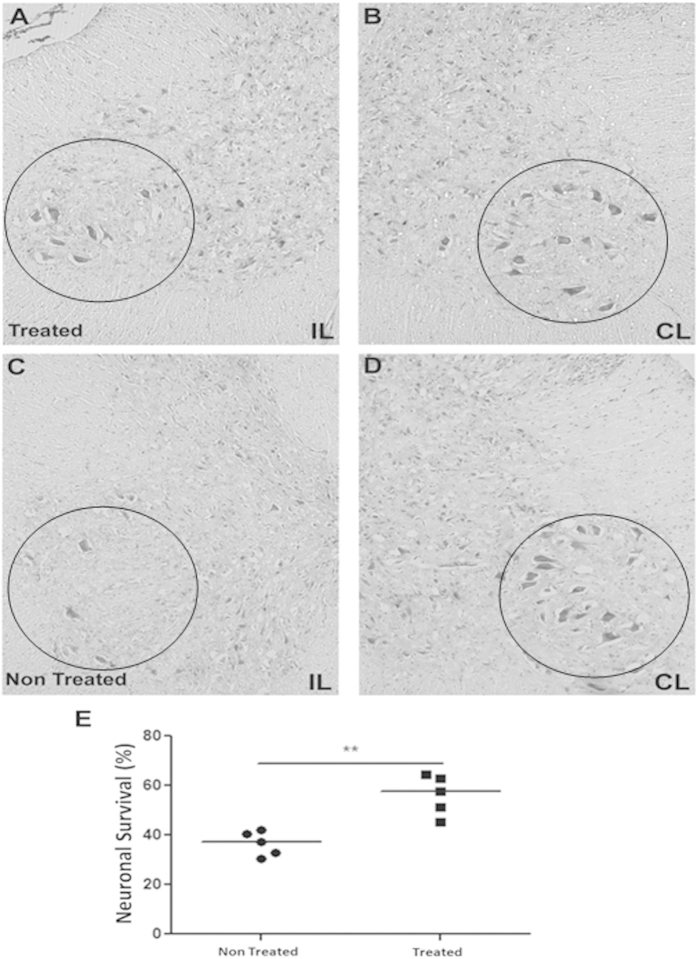
Neuronal survival two weeks after VRA and AT-MSCs implant. (**A**–**D**) Representative images of motor neuron cell bodies in the nerve motor nucleus (10×). (**A**,**C**) Ipsilateral (IL) and (**B**–**D**) Contralateral (CL) sides of AT-MSCs treated and non-treated lesion, respectively (**E**) Percentage of spinal motor neuron survival after PBS and AT-MSCs treatment. After AT-MSCs treatment, a higher number of motor neurons were capable to survive in the lesion (**p < 0.01).

The neuroprotective effect of soluble factors, such as BDNF and GDNF, has been well described. Confirming these findings, we showed that AT-MSCs expressed both factors (Fig. 1A). To quantify the protection of AT-MSCs treatment on motor neurons submitted to axotomy, the number of alpha motor neurons was counted on lesioned (Ipsilateral - IL) and non-lesioned (Contralateral - CL) sides of spinal cord, two weeks after avulsion (Fig. 3). No statistical differences were observed regarding the number of motor neurons on the Contralateral side of both groups (Table 1), and neuronal survival was represented as the percentage of the Contralateral side (Fig. 3B,D).

There was a greater number of surviving neurons with milder signs of chromatolysis in the treated group (Fig. 3A). Contrarily, there was intense motor neuron degeneration in the spinal cord motor nucleus of untreated animals (Fig. 3C), showing pyknotic nucleus and intense chromatolysis. Therefore, the number of surviving motor neurons on the ipsilateral was 50% higher for the treated than for the untreated group (treated = 56.25% ± 8.04; untreated = 36.66 ± 4.83%; mean ± SD; p = 0.0079; Fig. 3E).

**Figure 4:**
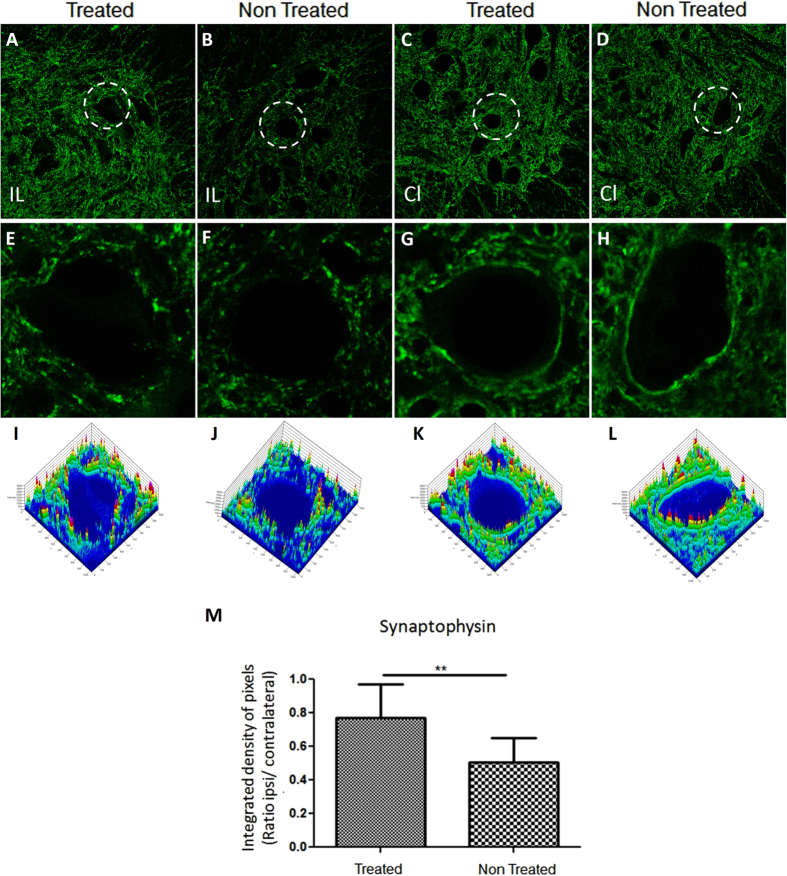
Representative image of synaptophysin immunolabelling in the spinal cord ventral horn after 2 weeks of lesion. ((**A**) 10×) The Ipsilateral (IL) of the treated group has stronger synaptophysin labeling than ((**B**) 10×) the Ipsilateral of non-treated group. ((**C**,**D**) 10×) The Contralateral of both groups showed similar synaptophysin immunolabeling pattern. (**E**–**H**) A representative motor neuron image analyzed by Start LSM Image Examiner (Carl Zeiss AIM Software) at zoom (5×). On surface of motor neuron at (**E**) the Ipsilateral of the treated group, the synaptophysin immunolabeling are better p reserved when compared to (**F**) Ipsilateral non-treated-group moto neuron, but less preserved than the (**G**,**H**) Contralateral motor neuron of both groups. (**I**–**L**) The Integrated density of pixels obtained by Start LSM Image Examiner (Carl Zeiss AIM Software) emphasizes the difference among moto neurons. (**M**) Graph represents the quantification of the immunolabeling in all groups (**p < 0.01).

For synaptic activity analysis after avulsion, immunostaining of the motor neuron body, using anti-synaptophysin antibody, was performed (Fig. 4). Synaptophysin expression was similar on the Contralateral sides of both groups (Fig. 4C,D,G,H,K,L). Motor neuron cell surface marker was well defined. There was an intense reactivity on the surface of Contralateral neurons, suggesting a large number of presynaptic inputs of alpha motor neurons (Fig. 4G,H). Intensity pixel analysis confirmed normal integrity of presynaptic inputs around motor neurons (Fig. 4K,L). Contrary to the Contralateral side, the intensity of synaptophysin expression on the Ipsilateral side was drastically reduced on the surface of the axotomized motor neurons of both groups (Fig. 4A,B,E,F,I,J). The synaptophysin expression however, was different between the groups (Fig. 4A,B). In treated animals, the motor neuron surface showed less reduction of pre-synaptic inputs compared to the untreated group (Fig. 4E,F), resulting in a reduction of the synaptic elimination process. (Treated group (Rt) = 0.772 ± 0.200, mean ± SD; Untreated group = 0.507 ± 0,143; p = 0.0015; Fig. 4M).

### AT-MSCs Effects on Glial cells

**Figure 5:**
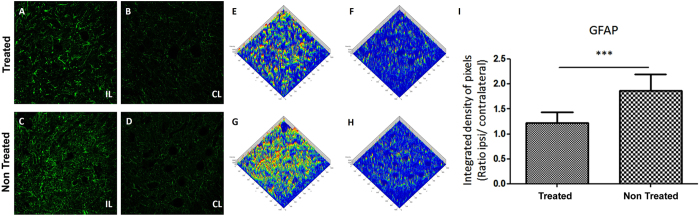
Representative image of Glial Fibrillary Acidic Protein (GFAP) in the spinal cord ventral horn after 2 weeks (10×). (**A**,**C**) The Ipsilateral of both groups has a stronger GFAP labeling than (**B**,**D**) the Contralateral. (**A**) The Ipsilateral of the treated group showed a distinguished reduction of astroglial reaction when compared to (**C**) non-treated group. (**E**–**H**) Integrated density of pixels, analyzed by Start LSM Image Examiner (Carl Zeiss AIM Software), points out the difference concerning to (**E**,**G**) Ipsilateral and (**F**,**H**) Contralateral of both groups and to Ipsilateral of (**E**) AT-MSCs and (**G**) PBS administration. (**I**) Graph represents the immunolabeling quantification in all groups (***p < 0.001).

**Figure 6:**
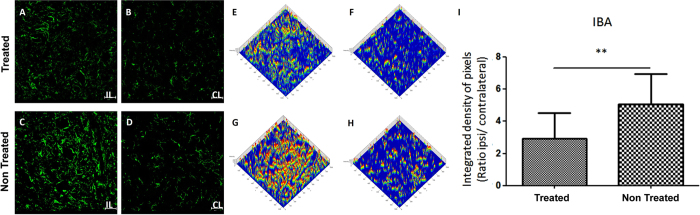
Representative image of Microglial activation by labelling of Ionized calcium binding adaptor molecule 1 (IBA1) in the spinal cord ventral horn after 2 weeks (10×). (**A**,**C**) The Ipsilateral side of both groups showed a higher IBA1 labelling than (**B**,**D**) the Contralateral. (**A**) The Ipsilateral side of the treated group showed a reduction of microglial activation when compared to (**C**) non-treated group. Integrated density of pixels analyzed by Start LSM Image Examiner (Carl Zeiss AIM Software), points out the difference concerning to (**E**,**G**) Ipsilateral and (**F**,**H**) Contralateral sides of both groups and to Ipsilateral (**E**) AT-MSCs and (**G**) PBS administration. (**I**) Graph represents the immunolabeling quantification in all groups (**p < 0.01).

Reactive astrogliosis and microglial activation were evaluated by anti-GFAP and anti-IBA1 staining respectively, two weeks after injury. There was a basal reactivity on the Contralateral side, in both groups, for both markers GFAP (astrocyte; Fig. 5) and IBA1 (microglia; Fig. 6). As expected, after injury there was an increased activity of astrocyte and microglia on the Ipsilateral side (Fig. 5A,C; Fig. 6A,C) compared to the Contralateral side (Fig. 5B,D; Fig. 6B,D). Therefore, the intensity of both glial markers (GFAP and IBA1) was lower in the treated than in the untreated group. The analysis by pixel intensity graphically represents the similarity of the intensity of GFAP and IBA1 labeling of both Contralateral side (Fig. 5F,H; Fig. 6F,H) and highlights the difference due to AT-MSCs treatment (Fig. 5E,G; Fig. 6E,G). Thus, AT-MSCs treatment reduced astrocyte (treated group = 1.227 ± 0.223, mean ± SD; untreated group = 1.870 ± 0.345; p = 0.0004; Fig. 5I) and microglia response to neuronal lesion (treated group = 2.697 ± 1.623, mean ± SD; untreated group = 5.094 ± 1.633p = 0.0032; Fig. 6I).

## Discussion

Recent studies suggest stem cell feasibility for treating central nervous system (CNS) diseases and injuries^43^,^44^,^45^, thus, we herein proposed the administration of Adipose Tissue Mesenchymal Stem Cells (AT-MSCs) following ventral root avulsion (VRA) in a human/rat xenograft model. From an experimental perspective, the possibility of using human stem cells in an animal model is a critical step before further clinical approaches, enabling the evaluation of possible immunoregulatory properties of these stem cells.

In the present study, AT-MSCs were extracted from lipoaspirate tissue treated by collagenase enzymatic digestion; the cells obtained were submitted to cell culture and characterization. AT-MSCs were adherent to plastic, they expressed CD90, CD73, CD105 and CD29 markers and lacked CD34, CD45 and HLA-DR markers. AT-MSCs were able to differentiate into three lineages (adipocytes, chondrocytes and osteocytes), according to MSC definition criteria^46^. Furthermore, transcripts showed neurotrophic factors (BDNF and GDNF) and anti-inflammatory cytokines (TGF-β1, HGF and IDO1) expression. AT-MSCs immunomodulatory ability on *in vitro* proliferation of lymphocytes T was also observed. The main novelty of this study is to show that human AT-MSCs prevent second phase neuronal injury, since they suppressed lymphocyte, astroglia and microglia effects, which finally contributed to rat motor-neuron survival and synaptic stability of the lesioned motor-neuron. Moreover, to our knowledge, our study showed, for the first time, the survival of the injected AT- MSCs for at least 14 days.

Fat tissue for mesenchymal stem cell acquisition is a good source of material for research and has a feasible potential for clinical use as human adipose tissue can easily be obtained in large quantities and does not present some of the common problems, such as low harvested cell number and limited tissue amount^47^,^48^,^49^. The effects of AT-MSCs cell therapy has also proved to be very beneficial in several animal models^22^,^50^,^51^,^52^,^53^, although positive effects are probably more dependent on the cytokines produced by AT-MSCs than on their ability to differentiate into other cell lineages^22^,^54^. These properties are also applied to mesenchymal stem cells obtained from the bone marrow (BM-MSCs)^50^,^55^.

Several studies indicate that AT-MSCs modulate the immune system negatively. In multiple sclerosis mouse models (EAE), Constantin and colleagues^56^ found that AT-MSCs administration during the preclinical phase of the disease exerted an anti-proliferative T lymphocyte effect. For type 1 diabetes model^26^, AT-MSCs treatment induced proliferation of regulatory T-cells, reducing Th1 and pro-inflammatory cytokine activity (IFN-γ, IL-2 and TNFα), whereas there was an increase in anti-inflammatory cytokines after 35 days of treatment. Peng and colleagues^57^ performed experiments with AT-MSCs and dendritic cells subjected to co-culture with CD4^+^ T cells and the group demonstrated that co-stimulatory molecules were downregulated (IL-12 and TNF); there was an increased IDO1 expression of dendritic cells and dendritic cells inhibited CD4^+^ T cells activation, confirming AT-MSCs immunomodulatory effect. Regarding human stem cell xenotransplant, review articles have shown that transplantation resulted in positive action for 7 immunocompetent recipient animal species^27^,^28^, these results are also associated with the low or absent HLA-DR expression on MSC´s membrane surface.

MSCs immunomodulatory effects seem to be mediated, in part, by the secretion of IDO-1 and anti- inflammatory cytokine expression^31^,^32^, as IL-10, TGFβ and HGF by themselves. IDO and anti-inflammatory cytokine release, mainly IL-10 and TGFβ, may enhance cells with regulatory proprieties diminishing the inflammatory response at the injury site^42^,^58^. Additionally, a number of studies have described the secretion of known neurotrophic factors by AT-MSCs, such as BDNF, GDNF, PDGF, FGF and others^59^,^60^,^61^,^62^, which lead to neuronal survival, axonal branching, synapse formation and differentiation of residing stem cells in the nervous system^56^. Thus, the main effects of AT-MSCs should occur through paracrine factor secretion. Despite the fact that the cells are localized at the injection site, as herein shown, we presume that the secretion of these factors by MSCs reaches the entire lesion area contributing to neuronal survival related to root avulsion.

Herein, human AT-MSCs immunomodulatory effect in a rat model (xenotransplantation) was observed. Human AT-MSCs were able to reduce *in vitro* T-lymphocyte proliferation, reduce *in vivo* T-cell activity in the injury area and survive for at least 14 days in the animal model (experimental period). Furthermore, in addition to HLA-DR absence on the surface of the cellular membrane, AT-MSCs expresses anti-inflammatory factors (TGFβ and HGF), and IDO1. T lymphocyte proliferation assay showed that the higher the AT-MSCs cell concentration, the lower T cell proliferation. Moreover, the presence of T lymphocyte (CD3) in the spinal cord section was reduced, suggesting that AT-MSCs acts through the paracrine effect, since the immunomodulatory action of these cells can also occur at a distance. Thus, results suggest that human AT-MSCs have a paracrine effect on the injured spinal cord microenvironment and the immunoregulatory effects of human AT-MSCs are apparently similar to animal AT-MSCs, when compared to other studies^26^,^56^,^63^. This is relevant clinical information for future allogeneic transplants.

Moreover, human AT-MSCs express neurotrophic factors, such as BDNF and GDNF, and the presence of these cells in the animal model may favor the survival of injured motor neurons, as in the control group most motor neurons had degenerated after 2 weeks. Furthermore, there was a reduction in astrogliosis and microglia activation, possibly reducing the effects of secondary damage caused by glial cells to motor neurons.

Despite GDNF effect being significantly more potent than BDNF in promoting axonal regeneration, the combined BDNF and GDNF treatment after tibial nerve section in adult rats has synergic effects and significantly increases the number of motor neurons that have regenerated their axons^64^. However, neurotrophic factors are not capable of maintaining the survival of affected motor neurons for long periods if there is no connection between the body and the target cell^65^. Thus, AT-MSCs therapy would be a strategy to allow neuronal survival and maintenance of spinal circuit (synaptophysin labelling preservation) for up to 2 weeks subsequent to root re-implantation.

Rodrigues and colleagues obtained similar results in a ventral root avulsion study after injection of allogeneic bone marrow MSCs (BM-MSCs)^60^, observing increased motor neurons survival, synaptic vesicle preservation and reactive astrogliosis reduction. In a comparative human AT-MSCs and BM-MSCs study in an animal model of spinal cord injury (SCI), Zhou and colleagues^14^ observed an increased angiogenesis at the lesion epicenter of the spinal cord, axonal recovery and reduction of inflammatory infiltrates, however the results were better for AT-MSCs treatment, also proving the human cell effects on the animal system and corroborating our results.

Despite our promising results, the development of a complete regenerative process and eventual recovery of the animal’s movements would necessarily involve re-implantation of avulsioned nerve roots. Recently, Espin and colleagues^13^ reported that root re-implantation combined with allogeneic injection of BM-MSCs had synergistic neuroprotective effects and increased growth of axonal regeneration. Therefore, further studies are required to assess the regenerative potential of avulsed motor neurons subjected to human AT-MSCs treatment and root re-implantation, as AT-MSCs appear to be a better source regarding abundance, accessibility and results.

Lastly, the present work combined morphological and immune evaluation of human AT-MSCs properties in an animal model of proximal lesion of spinal cord motor roots. Immunomodulatory effects showed to be combined with the neuroprotective profile of human AT-MSC, suggesting that the proposed therapy is feasible in clinical studies for autologous and allogeneic stem cell injection.

## Additional Information

**How to cite this article**: Ribeiro, T. B. *et al.* Neuroprotection and immunomodulation by xenografted human mesenchymal stem cells following spinal cord ventral root avulsion. *Sci. Rep.* **5**, 16167; doi: 10.1038/srep16167 (2015).

## Supplementary Material

Supplementary Information

## Figures and Tables

**Table 1:** Differentiation Medium and Cell Staining.

	Differentiation Medium	Cell Staining
Osteogenic differentiation	DMEM (Invitrogen)	Alizarin Red S
	10% FBS (Invitrogen)	
	0.1 μM dexamethasone (Sigma)	(Sigma)
	50 μM ascorbate-2-phosphate (Sigma)	
	10 mM beta-glycerophosphate (Sigma)	
Adipogenic differentiation	DMEM	Oil-Red O
	10% FBS	
	0.5 mM isobutylmethylxanthine (IBMX - Sigma)	(Sigma)
	1 μM dexamethasone (Sigma)	
	10 μM insulin (Sigma)	
	200 μM indomethacin (Sigma)	
Condrogenic differentiation	DMEM	Syrus red, resorcin and fuchsin
	100 ng/ml transforming growth factor - β3 (TGF-β3 - Peprotech)	
	100 nM sodium pyruvate (Sigma)	
	1 mM of dexamethasone (Sigma)	(Sigma)
	50 nM of ascorbic acid (Sigma)	
	0.5X insulin-transferrin-selenium A (ITS-A - Invitrogen)	
	0.,2% human albumin (Sigma)	

**Table 2:** Primers for PCR.

Gene	Sequence	Productlength
BDNF	FW: 5′ GGGCAAACACTGCATGTCTCTGGT 3′	131
	RV: 5′ TCCAGGCCATTCTGCAGGGTCA 3′	
GDNF	FW: 5′ CGCCCTTCGCGCTGAGCA 3′	145
	RV: 5′ CGCTCTCTTCTAGGAAGCACTGCCA 3′	
TGF-β1	FW: 5′ GCGTGCTAATGGTGGAAACC 3′	100
	RV: 5′ GCTTCTCGGAGCTCTGATGTG 3′	
HGF	FW: 5′ TGACTCCGAACAGGATTCTTTCA 3′	101
	RV: 5′ GCAGGGCTGGCAGGAGTT 3′	
IDO1	FW: 5′ TTGGAGAAAGCCCTTCAAGTG 3′	100
	RV: 5′ TGCCTT TCCAGCCAGACA A 3′	
Β2-Microglobulina	FW: 5′ ATGTCTCGCTCCGTGGCCTTAGCT 3′	375
	RV: 5′ CCTCCATGATGCTGCTTACATGTC 3′	

**Table 3:** Absolute mean number of neurons sampled per section in the ipsilateral and contralateral sides of the spinal cord in the different experimental groups (mean ± SD).

	Experimental Groups			
	Treated (n = 5)		Non-treated (n = 5)	
	Ipslateral	Contralateral	Ipslateral	Contralateral
Numbers of neurons per section	8.19 ± 2.10	14.04 ± 2.04	4.78 ± 1.31	13.71 ± 2.17
Numbers corrected by Abercrombie’s formulas	7.99 ± 2.,07	13.71 ± 1.97	4.74 ± 1.35	13.63 ± 2.30
