# Supplementary material for: Neuroprotection and immunomodulation by xenografted human mesenchymal stem cells following spinal cord ventral root avulsion
Source: Sci Rep. 2015 Nov 9;5:16167. doi: 10.1038/srep16167 (PMC4637826; doi:10.1038/srep16167)
Supplement: Supplementary Information [file srep16167-s1.pdf]

Neuroprotection and immunomodulation by xenografted human mesenchymal stem cells following spinal cord ventral root avulsion

Thiago B. Ribeiro[1], Adriana S.S. Duarte[1], Ana Leda F. Longhini[1,2], Fernando Pradella[2], Alessandro S. Farias[2], Angela C.M. Luzo[1], Alexandre L. R. Oliveira[3], Sara Teresinha Olalla Saad[1].

[1]Hematology and Hemotherapy Center-University of Campinas/Hemocentro-Unicamp, Instituto Nacional de Ciência e Tecnologia do Sangue, Campinas, São Paulo, Brazil

[2]Neuroimmunomodulation Group, Dept. Genetics, Evolution and Bioagents – University of Campinas – Campinas –Brazil

[3]Dept. of Structural and Functional Biology - Institute of Biology - University of Campinas – Campinas –Brazil

Running title: Neuroprotection and immunomodulation by xenografted human mesenchymal stem

Key words xenografted, mesenchymal stem cells, immunomodulation,

Corresponding Author:

Sara Teresinha Olalla Saad, MD, PhD

Hematology and Hemotherapy Center, University of Campinas

Rua Carlos Chagas, 480, CEP 13083-878

Campinas, SP, Brazil

Phone: 55-19-35218734; Fax: 55-19-3289-1089

E-mail: sara@unicamp.br

Supplement Figure 1 – Human AT-MSC grafting in rat spinal cord. (A) Schematic figure of the surgery and injection procedure. (B) Injection site of AT-MSC (arrow) in the spinal cord ventral horn submitted to avulsion (10x) (C) Qdot 655 labelled AT-MSC (red) with qdot655 (D) merge figure showing the presence of AT-MSC in the injection site area (20x).

Supplement Figure 2 – Representative figures and characterization of AT-MSC at 4th passage. (A) Antigen membrane surface characterized by flow cytometry shows a typical expression pattern of MSC. Cells express CD90, CD73, CD105, CD29 and HLA-ABC and lack expression of CD34, CD45 and HLA-DR. (B) AT-MSC is plastic adherent and present fibroblast morphology. AT-MSC were differentiated into mesodermal lineage, such as (C) osteogenic lineage characterized by calcium deposition (Red S Alzarin staining); (D) adipogenic lineage characterized by lipid vesicles (Oil Red O staining); and (E) chondrogenic lineage characterized by glycosaminoglycan matrix production (Alcian blue staining).

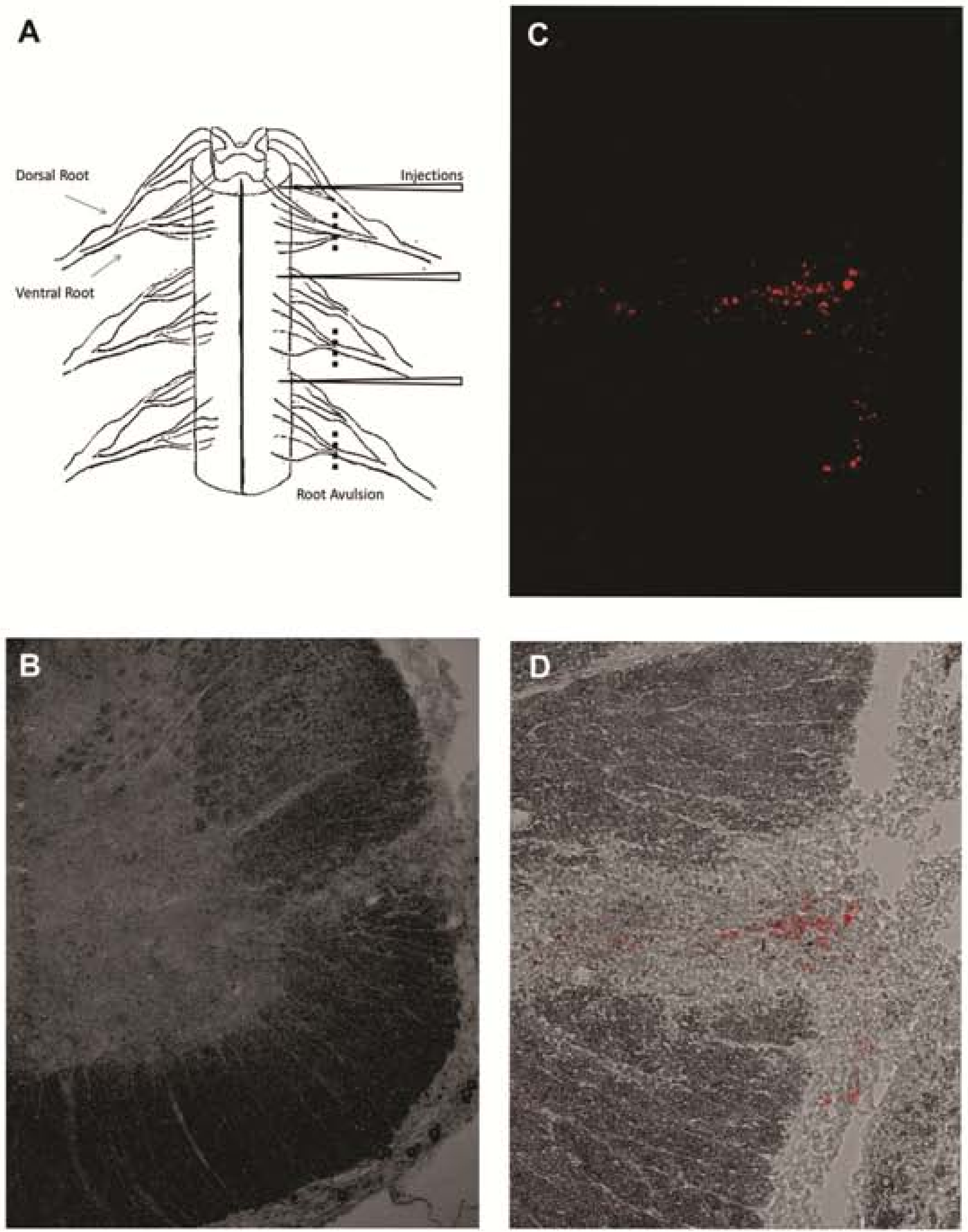
A
Dorsal Root
Ventral Root
Injections
Root Avulsion
B
C
D

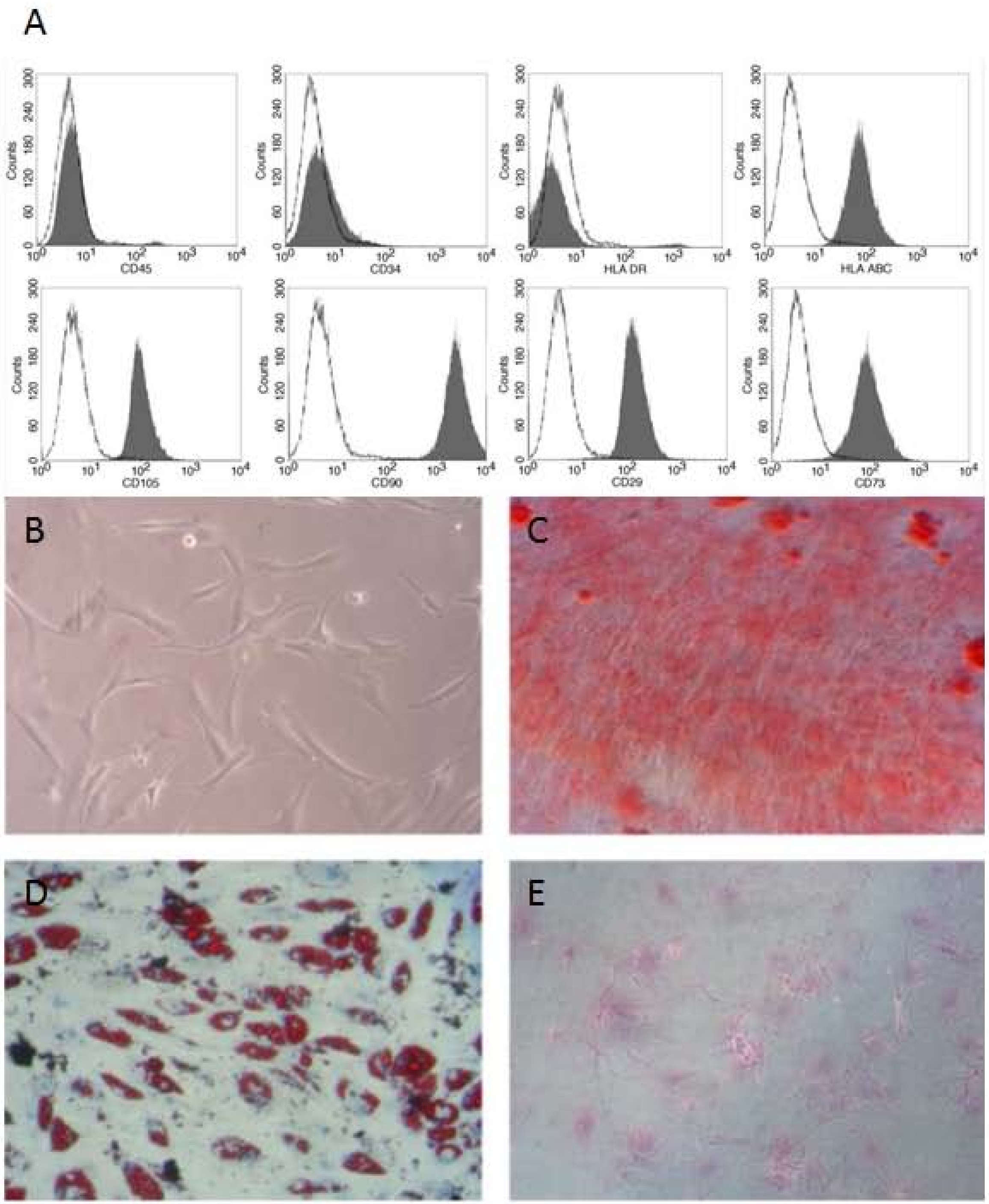
A
CD45
CD34
HLA DR
HLA ABC
CD105
CD90
CD29
CD73
Counts
B
C
D
E
